# Haplogroup Prediction Using Y-Chromosomal Short Tandem Repeats in the General Population of Bosnia and Herzegovina

**DOI:** 10.3389/fgene.2021.671467

**Published:** 2021-06-11

**Authors:** Naida Babić Jordamović, Tamara Kojović, Serkan Dogan, Larisa Bešić, Lana Salihefendić, Rijad Konjhodžić, Vedrana Škaro, Petar Projić, Vesna Hadžiavdić, Adna Ašić, Damir Marjanović

**Affiliations:** ^1^Department of Genetics and Bioengineering, International Burch University, Sarajevo, Bosnia and Herzegovina; ^2^ALEA Genetic Center, Sarajevo, Bosnia and Herzegovina; ^3^Molecular Anthropology Laboratory, Center for Applied Bioanthropology, Institute for Anthropological Research, Zagreb, Croatia; ^4^DNA Laboratory, Genos Ltd., Zagreb, Croatia; ^5^Department of Biology, University of Tuzla, Tuzla, Bosnia and Herzegovina

**Keywords:** Bosnia and Herzegovina, *in silico* haplogroup assignment, population genetics, Y chromosome, Y haplogroup, Y-short tandem repeats

## Abstract

Human Y-chromosomal haplogroups are an important tool used in population genetics and forensic genetics. A conventional method used for Y haplogroup assignment is based on a set of Y-single nucleotide polymorphism (SNP) markers deployed, which exploits the low mutation rate nature of these markers. Y chromosome haplogroups can be successfully predicted from Y-short tandem repeat (STR) markers using different software packages, and this method gained much attention recently due to its labor-, time-, and cost-effectiveness. The present study was based on the analysis of a total of 480 adult male buccal swab samples collected from different regions of Bosnia and Herzegovina. Y haplogroup prediction was performed using Whit Athey’s Haplogroup Predictor, based on haplotype data on 23 Y-STR markers contained within the PowerPlex® Y23 kit. The results revealed the existence of 14 different haplogroups, with I2a, R1a, and E1b1b being the most prevalent with frequencies of 43.13, 14.79, and 14.58%, respectively. Compared to the previously published studies on Bosnian-Herzegovinian population based on Y-SNP and Y-STR data, this study represents an upgrade of molecular genetic data with a significantly larger number of samples, thus offering more accurate results and higher probability of detecting rare haplogroups.

## Introduction

The paternally inherited non-recombining portion of the Y chromosome (NRY) is the material of choice when it comes to tracing the paternal lineage of populations (Jobling and Tyler-Smith, 2003; Butler, 2012; Felkel et al., 2019). The potential applications of the NRY in human population studies are numerous and it is, therefore, extensively used in the studies of human origin, population history, sex-biased admixture, male-female differences in migration, and medical and clinical studies (Jobling and Tyler-Smith, 2003; Butler, 2005; Marjanović et al., 2018). The two most important classes of Y-chromosomal markers deployed in such studies are short tandem repeats (Y-STRs) and single nucleotide polymorphisms (Y-SNPs), and as these markers are transmitted directly by paternal lineage without recombination, they are highly sensitive to genetic drift and allow for a very informative haplotype construction (Butler, 2012; Mahal and Matsoukas, 2018).

A single Y haplogroup represents a group or a family of Y chromosomes related by descent or ancestry, and each such haplogroup is determined by a specific set of Y-SNPs, which makes them extremely important when it comes to better understanding of past migrations and demographic processes that shaped modern populations (Marjanović et al., 2006). Since Y-SNP detection and analysis is costly, time-consuming, labor intensive, and multiple markers are required to be analyzed prior to final determination of an individual’s haplogroup, innovative approaches have recently been investigated. Indeed, these approaches are mainly related to Y haplogroup prediction using a set of Y-STR markers or a haplotype (Dogan et al., 2016b).

**Figure 1:**
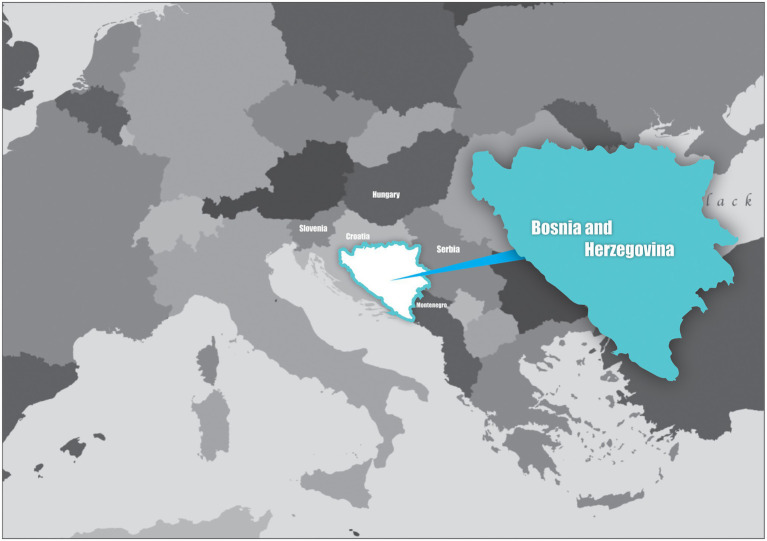
Geographical position of Bosnia and Herzegovina.

Population genetic studies are of high importance in Bosnia and Herzegovina (B&H; Figure 1) as they are suitable for the detection of all changes in this relatively small population and offer a new insight into the current population structure (Marjanović et al., 2005, 2006). Numerous archeological artifacts are proving that the territory of modern B&H has been inhabited since the Neolithic era (Malcolm, 1996). Interestingly, some of them imply that the first inhabitants settled in the area as late as the Paleolithic era (Marjanović et al., 2005). Later, during the Early Bronze Age, different Illyrians populations had settled in the various B&H region (Wilkes, 1995), and the Romans governed those tribes for more than 5 centuries (Klaić, 1990). As a result, a significant number of Roman soldiers have settled down in the area (Malcolm, 1996). After the fall of the Roman Empire, as the borderline between the Eastern and Western empires, this region was massively invaded by various tribes, such as the Goths Avar, the Slavs, and others. Additionally, expansion of the Ottoman Empire into this part of the Balkans in the fifteenth century left important cultural but also demographical impact on the modern B&H (Pirić et al., 2020). All these historical episodes created a fascinating genetic diversity within the modern multiethnic and multi-religious B&H society. Nowadays, B&H is a small country with a stormy history. Therefore, it is not surprising that the modern B&H population is one of the most genetically studied areas.

A previously published study observing SNPs was published using 256 samples (Marjanović et al., 2005). A later study focusing on the 23 Y-STR loci was published on a minimum number of 100 samples (Dogan et al., 2016a). Therefore, the main goal of this research was to investigate if significantly increasing the examined population size (compared with the previous studies) would alter the established balance between the most common haplogroups in B&H, and identify the presence of rare, previously not detected Y-haplogroups within this region.

## Materials and Methods

The study is based upon the collection of haplotype data from 480 unrelated male individuals who voluntarily participated in the project *The Ancestors in us – Genetic Heritage of Bosnia and Herzegovina* (Bosnian: *Preci u nama – Genetičko blago Bosne i Hercegovine*), designed and implemented by Mladinska knjiga (Sarajevo, B&H; Marjanović et al., 2019). The samples were collected by the participants according to the usual procedure for the collection of undisputed samples of buccal mucosa using a sterile cotton swab. Instructions for proper sampling were delivered to each participant with the ordered kit, and additional video instructions were published on the project web page. Each respondent signed a form of voluntary consent to participate in this research and to agree that the anonymous population data will be published. The study was conducted in accordance with the Helsinki Declaration on research involving human participants.

Genomic DNA was isolated using Qiagen DNeasy™ Blood and Tissue Kit (Hilden, Germany) according to manufacturer’s recommendations, while DNA quantification was assessed on Qubit® 2.0 fluorometer (Thermo Fisher Scientific, Waltham, MA, United States). PCR amplification of target 23 Y-STR loci was performed using PowerPlex® Y23 System (Promega Corporation, Madison, WI, United States) following manufacturer’s recommendations. Amplicons were detected on an ABI PRISM® 310 Genetic Analyzer (Applied Biosystems, Foster City, CA, United States). Raw data analysis was performed using ABI PRISM® Data Collection software, while the final Y-STR DNA profiles were generated using GeneMapper® ID v3.2 software. Allele frequencies and haplotype data obtained in this way are currently submitted for publication elsewhere (Babić Jordamović et al., unpublished data).

Y-chromosomal haplogroup prediction using allele frequencies on 23 Y-STR loci was performed using Whit Athey’s Haplogroup Predictor v5, an algorithm based on the Bayesian-allele-frequency approach (Athey, 2006, 2013).

## Results and Discussion

**Figure 2:**
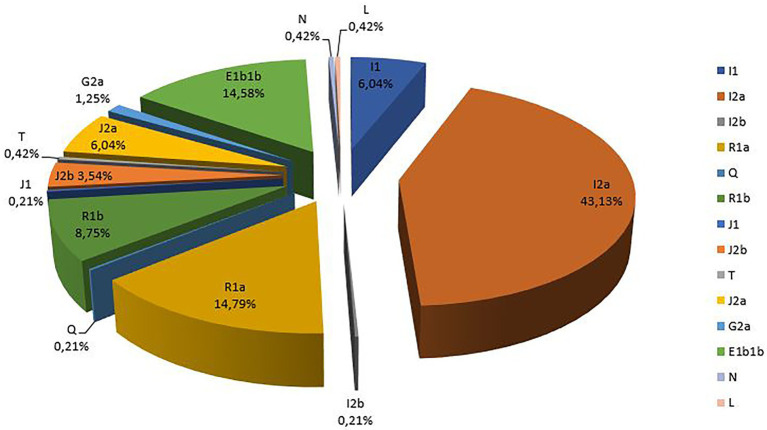
Prevalence of Y-chromosomal haplogroups in the population of Bosnia and Herzegovina (*n* = 456).

The results of Y haplogroup prediction using Whit Athey’s Haplogroup Predictor program are summarized in Figure 2. According to our previously published results (Dogan et al., 2016b) using the Whit Athey’s software, in comparison with other predictors, this software proved to be the most valuable tool for *in silico* Y haplogroup assignment from Y-STR data even in relatively small and heterogeneous populations such as the B&H population. Therefore, we have decided to use this tool in this study. Additionally, for the samples where prediction accuracy was lower than 90%, we have used the NevGen algorithm (Cetkovic Gentula and Nevski, 2015) as a verification method. Successful haplogroup assignment was obtained for all 480 Y-STR profiles. The Y chromosomal STR data of the present study were submitted to Y-STR Haplotype Reference Database (YHRD) and the accession number YA003787 was assigned.^1^

Prediction accuracy was estimated to be 100% in 456 cases. For the remaining 24 samples (5%), prediction accuracy varied between 52.2 and 99.9% (Table 1). Out of a total of 14 detected haplogroups, the most prevalent was I2a, which accounts for 43.13% of all samples, followed by R1a (14.79%) and E1b1b (14.58%). The remaining eight haplogroups were significantly less frequent.

**Table 1:** The list of haplotypes for which Y haplogroup prediction accuracy was lower than 100%.

Haplotype	Whit Athey’s prediction	Probability (%)
Sample 1	R1b	52.2
Sample 2	Q	53.6
Sample 3	J2b	59.9
Sample 4	I2a	64.7
Sample 5	I1	69.4
Sample 6	J2a	73.8
Sample 7	E1b1b	78.8
Sample 8	I1	89.4
Sample 9	J2a	91.2
Sample 10	G2a	97.6
Sample 11	R1b	97.9
Sample 12	J2b	98.2
Sample 13	L	99
Sample 14	J2a	99.5
Sample 15	J2a	99.5
Sample 16	I1	99.6
Sample 17	J2a	99.7
Sample 18	E1b1b	99.8
Sample 19	J2b	99.8
Sample 20	J2b	99.8
Sample 21	J1	99.9
Sample 22	J2b	99.9
Sample 23	J2b	99.9
Sample 24	I1	99.9

High prevalence of haplogroup I with its sublineage I2a (43.13%) was expected, when considering previously published literature (Marjanović et al., 2005; Dogan et al., 2016a). In three main ethnic groups in B&H, I2a accounted for 71% of all haplogroups in Croats, 44% in Bosniaks, and 31% in Serbs (Marjanović et al., 2005) based on Y-SNP analysis. In the previous study of Croatian and Serbian populations, sublineage I2a was also the most frequent (Barać et al., 2003; Peričić et al., 2005; Kačar et al., 2019). Paleolithic origin of this haplogroup suggests the possibility of modern population expansion from one of the post-Glacial refuges into the rest of the Balkan Peninsula (Marjanović et al., 2006). It is believed that haplogroup I arrived in the area of Balkan Peninsula around 25,000 years ago from the Middle East through Anatolia (Battaglia et al., 2009; Primorac et al., 2011). However, recent insights into this research area suggest the possibility of this haplogroup being associated with more recent population movements; however, this requires additional analyses (Marjanović et al., 2019). In comparison to other European populations, I2a could be considered a typical Southeast European haplogroup (Kushniarevich et al., 2015).

Haplogroup R, with its major sublineage R1a, was the second most abundant in the study population of B&H with frequency of 14.79%. In the previous Y-SNP-based study, the haplogroup R accounted for around 14% of all Y chromosomes with an even distribution among three ethnic groups, namely 15% in Bosniaks, 14% in Serbs, and 12% in Croats (Marjanović et al., 2005). In the total Serbian population, haplogroup R was found in a frequency of 15.9%, while it was shown to be more common in Croatia accounting for a total of 33.9% in the mainland population. In the Slovenian population, the prevalence of this haplogroup was 37% (Barać et al., 2003; Peričić et al., 2005; Kačar et al., 2019). Theories on R1a origins suggest the flow of haplogroup R from West Asia into the Balkan region as a post-Last Glacial Maximum (LGM) event, that is, during the Mesolithic time (Myres et al., 2011; Primorac et al., 2011). The connection between haplogroups I and R was described through migration and gene flow between Europe and Middle East using both autosomal and Y-chromosomal markers (Kovačević et al., 2014). In contrast, subclade R1b occurred in a much lower frequency in our population with a prevalence of 8.75%. Similar results were obtained in the previous study of the population of B&H based on 100 Y-STR profiles, whereby R1b accounted for only 4% of the total (Dogan et al., 2016a).

Haplogroup E was detected in the present study through its sublineage E1b1b (14.58%), which is characteristic for European male individuals (Primorac et al., 2011). The previously studied population of B&H marked E1b1b as the second most prevalent haplogroup in the population with a frequency of 13.7% based on Y-SNP analysis (Marjanović et al., 2005, 2006). The similarity in frequency of E1b1b and R1a in this study population of B&H was also shown in the Y-STR-based study, whereby these two haplogroups appear in the frequency of 17% each (Dogan et al., 2016a). This haplogroup was found to be less frequent in the population of Croatia (5.5%) when compared to the total Serbian population (18.2%; Barać et al., 2003; Peričić et al., 2005; Kačar et al., 2019). Regarding its origins, one line of evidence suggests that E1b1b arrived in Europe during the Neolithic time as a post-LGM event from Asia and Africa, while the other opinion is that this haplogroup is Balkans-specific and that it originated around 8,000 years ago as a consequence of Greek colonization toward the northern part of the Peninsula (Battaglia et al., 2009; Primorac et al., 2011).

Rare haplogroups N, L, and T (each present in 0.42% of all samples), as well as Hg Q (with frequency of 0.21%) were detected in this study, and are mainly uncharacteristic for the area of the Balkans as they were not detected previously. In fact, haplogroups L, T, and Q were detected for the first time in the B&H population. Haplogroup N has most probably expanded from Northern Eurasia to Eastern Europe and is present in low frequencies in the Balkans and Central Asia (Ilumäe et al., 2016). Previously published research on 100 Y-STR haplotypes recorded Hg N in frequency of 1% (Dogan et al., 2016a), but it was not detected in the Y-SNP study (Marjanović et al., 2006). Haplogroup L is associated with South Asia and India but is also found in low frequencies in Central Asia, Southwest Asia, and Southern Europe. With its alternative phylogenetic name K1a, haplogroup L is closely related to haplogroup T (Mahal and Matsoukas, 2018). Haplogroup T, also known by its phylogenetic name K1b, possibly originated in Western Asia and spread to East Africa, South Asia, and Southern Europe (Mendez et al., 2011; Mathieson et al., 2018). Finally, haplogroup Q represents the only Pan-American haplogroup, confirms the Asian origin of Native Americans, and provides an insight into the main Asian-American migrations (Grugni et al., 2019).

Additionally, analysis of 23 Y-STR loci on the currently studied population of B&H (unpublished data) quantified through *R*st values and calculated based on the results of the same set of markers from other populations from the YHRD database, indicates that the Bosnian-Herzegovinian population does not deviate significantly from the neighboring populations of Serbia, Croatia, and Slovenia, while it stands out drastically from the populations of Belgium, Italy, Germany, Austria, and the Czech Republic (Babić Jordamović et al., unpublished data).

The current study performed on a significantly increased sample size generally confirmed the previous main haplogroup balance. However, it offered a more detailed understanding into the genetic diversity of the population of B&H and provided insight into rare and unusual haplogroups. This includes L, T, and Q, which have been detected as part of the region’s population genetic diversity for the first time. Therefore, it can be concluded that this study improves the current findings regarding the population and presents this information in a more reliable way.

## Data Availability Statement

The datasets presented in this study can be found in online repositories. The names of the repository/repositories and accession number(s) can be found at: https://yhrd.org/, YA003787.

## Ethics Statement

Ethical review and approval was not required for the study on human participants in accordance with the local legislation and institutional requirements. The patients/participants provided their written informed consent to participate in this study.

## Author Contributions

NJ and TK participated in laboratory work, data analysis, and manuscript drafting. SD conducted data analysis and visual representation of the data. LB, LS, RK, VŠ, and PP performed laboratory work and data analysis. VH participated in study design. AA participated in laboratory work, data analysis, and manuscript preparation. DM conceived the research idea, designed the work, and participated in data analysis and manuscript preparation. All authors contributed to the article and approved the submitted version.

### Conflict of Interest

LS and RK were employed by the company ALEA Genetic Center (AGC) in Sarajevo, Bosnia and Herzegovina. VŠ and PP were employed by the company Genos Ltd. in Zagreb, Croatia.The remaining authors declare that the research was conducted in the absence of any commercial or financial relationships that could be construed as a potential conflict of interest.

## Footnotes

^1^www.yhrd.org
